# Vaginal stenosis and dilator use after pelvic brachytherapy

**DOI:** 10.15649/cuidarte.3309

**Published:** 2024-07-11

**Authors:** Luciana Martins da Rosa, Maria Eduarda Hames, Mirella Dias, Luciana Puchalski Kalinke, Claudia Manuela Siqueira de Oliveira, María Angélica Arzuaga-Salazar

**Affiliations:** 1 Universidade Federal de Santa Catarina, Florianópolis, Santa Catarina, Brazil. E-mail: luciana.m.rosa@ufsc.br Universidade Federal de Santa Catarina Universidade Federal de Santa Catarina Florianópolis Santa Catarina Brazil luciana.m.rosa@ufsc.br; 2 AC Camargo Cancer Center, Sâo Paulo, Sâo Paulo, Brazil. E-mail: maria.hames@accamargo.org.br A.C. Camargo Cancer Center AC Camargo Cancer Center Sâo Paulo Sâo Paulo Brazil maria.hames@accamargo.org.br; 3 Centro de Pesquisas Oncológicas, Florianópolis, Santa Catarina, Brazil. E-mail: mirella.dias@posgrad.ufsc.br Centro Brasileiro de Pesquisas Físicas Centro de Pesquisas Oncológicas Florianópolis Santa Catarina Brazil mirella.dias@posgrad.ufsc.br; 4 Universidade Federal do Paraná, Curitiba, Paraná, Brazil. E-mail: kalinkeluciana@gmail.com Universidade Federal do Paraná Universidade Federal do Paraná Curitiba Paraná Brazil kalinkeluciana@gmail.com; 5 Universidade Federal de Santa Catarina, Florianópolis, Santa Catarina, Brazil. E-mail: claudiamanuela0780@gmail.com Universidade Federal de Santa Catarina Universidade Federal de Santa Catarina Florianópolis Santa Catarina Brazil claudiamanuela0780@gmail.com; 6 Facultad de Enfermería. Universidad de Antioquia. Medellín, Antioquia, Colombia. E-mail: maria.arzuaga@udea.edu.co Universidad de Antioquia Facultad de Enfermería Universidad de Antioquia Medellín Antioquia Colombia maria.arzuaga@udea.edu.co

**Keywords:** Constriction, Pathologic, Genital Neoplasms, Female, Brachytherapy, Nursing, Physical Therapy Department, Hospital, Constricción Patológica, Neoplasias de los Genitales Femeninos, Braquiterapia, Enfermería, Servicio de Fisioterapia en Hospital, Constrigao Patológica, Neoplasias dos Genitais Femininos, Braquiterapia, Enfermagem, Servigo Hospitalar de Fisioterapia.

## Abstract

**Introduction:**

To prevent vaginal stenosis, the use of a vaginal dilator is recommended.

**Objective:**

To analyze sociodemographic data, gynecological conditions and the use of vaginal dilator after pelvic brachytherapy.

**Materials and Methods:**

Cross-sectional, retrospective study, period 2016-2020, collected between October/2020 and February/2021, from records of women with gynecological cancer treated with brachytherapy at the Centro de Pesquisa Oncológicas (Brazil). The variables included sociodemographic data and gynecological conditions in following the treatment. In the analysis, descriptive statistics, chi-squaretest, Fisher's exact test and Mann-Whitney test were applied.

**Results:**

519 patients records were included in the investigation; the analyzes showed significant associations between the topography and staging (p<0.001), education (p=0.004) and age (p<0.001); the comparison between the distribution of the ionizing radiation dose showed a difference with the continued sexual relationship category (p=0.006); the comparison between the proportions ofcontinued sexual relationship and using a vaginal dilator was significant (p<0.001); 49.10% (131) adhered to the use of vaginal dilator; 24.50% (127) are not sexually active and do not adhere to the use ofthe dilator.

**Discussion:**

It is evident that social and gynecological conditions interfere with the presence of vaginal stenosis and the use of a vaginal dilator after pelvic brachytherapy.

**Conclusions:**

The adherence found in the use of dilator affirms the contributions and the need for health education by nurses and physicaltherapists during and following the treatment.

## Resumen

**Introducción:**

Para prevenir la estenosis vaginal se recomienda el uso de un dilatador vaginal.

**Objetivo:**

Analizar datos sociodemográficos, condiciones ginecológicas y el uso de dilatadores vaginales después de la braquiterapia pélvica.

**Materiales y Métodos:**

Estudio transversal, retrospectivo, período 2016-2020, recolectado entre octubre/2020 y febrero/2021, en historias clínicas de mujeres con cáncer ginecológico tratadas con braquiterapia en el Centro de Investigaciones Oncológicas (Brasil). Las variables cubrieron datos sociodemográficos y condiciones ginecológicas posteriores al tratamiento. En el análisis se aplicó estadística descriptiva, prueba de chi-cuadrado, prueba exacta de Fisher y prueba de Mann-Whitney.

**Resultados:**

Se incluyeron en la investigación 519 historias clínicas; los análisis mostraron asociaciones significativas entre topografía y puesta en escena (p<0,001), educación (p=0,004) y edad (p<0,001); la comparación entre la distribución de la dosis de radiación ionizante mostró diferencia con la categoría que mantenía relaciones sexuales (p=0,006); la comparación entre las proporciones de mantener relaciones sexuales y utilizar dilatador vaginal fue significativa (p<0,001); el 49,10% (131) adhirieron al uso del dilatador vaginal; El 24,50% (127) no mantuvo relaciones sexuales y no adhirió al uso del dilatador.

**Discusión:**

Es claro que las condiciones sociales y ginecológicas afectan la presencia de estenosis vaginal y el uso del dilatador vaginal después de la braquiterapia pélvica.

**Conclusiones:**

La adherencia encontrada en el uso del dilatador confirma los aportes y la necesidad de educación en salud por parte de enfermeros y fisioterapeutas durante y después del tratamiento.

## Resumo

**Introdujo:**

Para prevenir a estenose vaginal é recomendado o uso de dilatador vaginal.

**Objetivo:**

Analisar dados sociodemográficos, condigóes ginecológicas e o uso do dilatador vaginal após a braquiterapia pélvica.

**Materiais e Métodos:**

Estudo transversal, retrospectivo, período 2016 2020, coletado entre outubro/2020 e fevereiro/2021, em prontuários de mulheres com cáncer ginecológico tratadas com braquiterapia no Centro de Pesquisas Oncológicas (Brasil). As variáveis abrangeram dados sociodemográficos e condigóes ginecológicas no seguimento do tratamento. Na análise aplicou-se estatística descritiva, teste de qui-quadrado, Exato de Fisher e de Mann-Whitney.

**Resultados:**

519 prontuários foram incluidos na investigado; as análises mostraram associagóes significativas entre topografia e estadiamento (p<0,001), escolaridade (p=0,004) e idade (p<0,001); a comparado entre a distribuigao da dose de radiado ionizante mostrou diferenga com a categoria manutengao da relagao sexual (p=0,006); a comparagao entre as proporgóes da manutengao da relagao sexual e uso do dilatador vaginal foi significativa (p<0,001); 49,10% (131) aderiram ao uso do dilatador vaginal; 24,50% (127) nao mantiveram a relagao sexual e nao aderiram ao uso do dilatador.

**Discussao:**

Evidencia-se que as condigóes sociais e ginecológicas interferem na presenga da estenose vaginal e no uso do dilatador vaginal após a braquiterapia pélvica.

**Conclusóes:**

A adesao encontrada no uso do dilatador afirma as contribuigóes e a necessidade de educagao em saúde por enfermeiros e fisioterapeutas durante e no seguimento do tratamento.

## Introduction

Treatment of advanced gynecological cancers involves pelvic radiotherapy, including teletherapy and high dose rate brachytherapy (BATD). A side effect of the treatment is vaginal stenosis. Vaginal canal complications may be exacerbated by post-treatment ovarian insufficiency or menopausal status, resulting in further decreased lubrication and thinning of vaginal tissues^1^.

Vaginal stenosis resulting from BATD occurs, in most cases, three months after the end of treatment. It is commonly associated with sexual dysfunction, which has a negative impact on women's quality of life, posing a source of physical and psychological suffering^2^.

Some factors may contribute to the emergence of vaginal stenosis, such as age, total dose of ionizing radiation administered and area treated (anatomical location and tumor size). Studies show that 11% of women had grade 1 vaginal stenosis and 15% had grade 2^3^. Another study states an incidence rate of 22.70% of grade 3 vaginal stenosis, over a 20-month follow-up period^4^. Including all degrees of stenosis, the percentage of women with this toxicity is close to 22%. Furthermore, it is a multifactorial occurrence, which favors underreporting^2^. Pelvic pain is found in around 10% of women^5^.

To prevent vaginal stenosis, health monitoring and vaginal dilation become a necessity. At the Oncological Research Center (CEPON), an oncological institution in the state of Santa Catarina (Brazil), to prevent vaginal stenosis, nurses (during the BATD period) and physiotherapists (following the BATD) advise the continued use of the vaginal dilator after the end of the treatment (the service uses a penis-shaped silicone device, offered free of charge to all women at the end of BATD).

To ensure proper attention to these women, the CEPON Physiotherapy service implemented exclusive care following treatment (starting three months after the end of BATD). On an ongoing basis, the service reinforces the guidelines initiated by nurses during treatment, as well as treating possible pelvic, urinary and intestinal dysfunctions (urinary/fecal incontinence) resulting from radiotherapy.

The practice of vaginal dilation seems to be the best way to prevent vaginal stenosis induced by pelvic radiotherapy, however, women's adherence to this practice is one of the biggest challenges faced by professionals^6^. It is empirically observed that health education during treatment and post treatment follow-up allows for greater adherence, contradicting the results of a systematic review^7^^)^ which states the lack of follow-up or the high rate of women abandoning adherence to the use of the drug. vaginal dilator.

Regarding the use of the vaginal dilator, a study recorded adherence of 13.50% of patients two months after brachytherapy and 10.20% six months after^5^. In this context, the importance of preventing vaginal stenosis and new investigations to better understand the topic and care for women stands out. Therefore, the objective of this study is to analyze sociodemographic data, gynecological conditions and the use of the vaginal dilator after pelvic brachytherapy.

## Materials and Methods

Cross-sectional, retrospective study, with data collection carried out between October 2020 and February 2021, in medical records of women with gynecological cancer after completion of BATD at CEPON (Brazil), in records of the first follow-up consultation at the Physiotherapy service after completion of BATD, in the 2016-2020 timeframe. Women with completed complete treatment were included (three applications of ionizing radiation in hysterectomized women and four applications in non-hysterectomized women), completed within a period of 15 days, with complete or partial records (some records did not record all of the variables investigated, in these cases the variables were recorded as no information). Records of women under 18 were excluded. The collected data was organized into spreadsheets in the *Microsoft* Excel *Program* and exported to SPSS version 25 *software* for descriptive and inferential analysis. The primary data set is registered in the Mendeley Data^8^ public repository.

The study variables covered: age, education, topography of gynecological cancer diagnosis, cancer staging, dose/ gray (Gy) administered in teletherapy and dose/ Gy administered in BATD, number of teletherapy and BATD sessions/applications , presence and degree of vaginal stenosis, frequency of use of the vaginal dilator, reasons for not using the vaginal dilator, maintenance of sexual intercourse, changes in vaginal touch.

To present the staging, the Staging System of the *International Federation of Gynecology and Obstetrics* (FIGO). The degree of vaginal stenosis registered at the CEPON Physiotherapy Service is classified as: Grade 0: asymptomatic women, without stenosis; Grade 1: mild vaginal shortening or narrowing; Grade 2: vaginal narrowing or shortening, not interfering with the gynecological examination; Grade 3: vaginal narrowing or shortening interfering with the use of tampons, sexual activity or gynecological examination^9^. The dilation exercise recommended by CEPON nurses and physiotherapists includes the use of the vaginal dilator three times a week, 20 minutes of dilation each time, when the woman must remain lying down with the dilator inserted into the vaginal canal.

Categorical variables were represented by absolute and relative frequency. The chi-square test was used to verify the association. When significant, categories with a positive local association were studied. The variables were represented by mean and standard deviation and by median and interquartile range (median [P25; P75]). Using the Shapiro-Wilk normality test, it was decided to use the Mann-Whitney or Kruskal-Wallis test to compare the distributions of the categories studied. The significance level adopted was p=0.05. Discussions of the results were supported by up-to-date scientific publications linked to the subject of the investigation.

The study followed the provisions of Resolution 466/2012 of the National Health Council for research with human beings in Brazil. The opinions approving the development of the study are registered under numbers 4.050.347 (proponent), of May 26, 2020, and 4.133.605 (co-participant) of July 3, 2020. A Free and Informed Consent Form was applied.

## Results

In the period 2016-2020, at CEPON, 522 women diagnosed with gynecological cancer underwent their first follow-up consultation at the Physiotherapy Service after the end of BATD; three of these women did not complete all prescribed brachytherapy sessions (incomplete treatment). Therefore, 519 service records (100%) were eligible for this study.

Table 1 presents the comparison between the proportions of diagnoses and categorical variables studied. It was found that 76.30% of women (396) with cervical cancer with a higher incidence in stage II, elementary school, aged between 50-59 years; followed by women with cancer in the same topography, elementary school, aged between 40-49 years. Another notable result was the percentage of 27% found among women under 40 years old (18-39 years old) (Table 1).

Furthermore, there were significant associations between the variable topography and staging (p<0.001), education (p =0.004), age group (p<0.001). The older the age and the lower the level of education, the greater the risk of endometrial cancer and, for the topography of the cervix, the data were similar, however the age range was among younger women. Stage I was associated with endometrial topography, stage II with the cervix, stage III with the ovary and stage IV with the vagina. The education/high school education category was associated with vaginal cancer and higher education with ovarian and vaginal cancer. The <50 years of age category was associated with cervical cancer and the >60 years of age category was associated with endometrial cancer (Table 1).

**Table 1:** Comparisons between diagnoses. Florianopolis (S.C.), Brazil, 2021

Diagnosis					
Sociodemographic and clinical variables	Uterine lap (n=396)	Endometrium (n=114)	Ovary (n=2)	Vagina (n=7)	p-Value
Staging (n=495)					<0.001
I	7.80(30)	52.40(55) †	0(0)	0(0)	
II	47.00(180) †	15.20(16)	0(0)	20(1)	
III	36.30(139)	26.70(28)	100(2) †	40(2)	
IV	8.90(34)	5.70(6)	0 (0)	40(2) †	
No information	13	9	0	2	
Education (n=512)					0.004
Illiterate	4.80(19)	6.40(7)	0(0)	0(0)	
Elementary School	56.20(221)	60.90(67)	0(0)	0(0)	
High school	25.70(101)	20.90(93)	0(0)	57.10(4) †	
University education	13.20(52)	11.80(13)	100(2) †	42.90(3) †	
No information	3	4	0	0	
Age group (n=518)					<0.001
<40	107(27) †	3.50(4)	0(0)	16.70(1)	
40 - 50	99(25) †	6.10(7)	0(0)	33.30(2)	
50 - 60	100(25.30)	27.20(31)	50(1)	33.30(2)	
60 - 85	90(22.70)	63.20(72) †	50(1)	16.70(1)	
No information	0	0	0	1	

*p-Value: Fisher's Exact Test; t categories with positive local association are underlined.*

It should be noted that some of the variables shown in Table 2 present loss of information, due to the absence of some records in the medical records that prevented the variables from being crossed. These losses were found in other variables, as can be seen in the results presented in the subsequent tables, thus, calculations were made according to existing data.

BATD dose/ Gy distributions showed a difference between the categories of maintenance of sexual intercourse (p =0.006) (Table 2). The dose/ Gy distributions in women without sexual intercourse were statistically lower when compared to women who maintained sexual intercourse. Comparisons between the distributions between having or not stenosis were not significant. The average total radiation dose varied between 67.9 and 74 Gy (Table 2).

**Table 2:** Comparison of the number of sessions and radiation dose with the maintenance of sexual intercourse and vaginal stenosis. Florianopolis (S.C.), Brazil, 2021. Median [P25; P75]

Radiation dose	n	Yes	No	p-Value
Maintenance of sexual intercourse				
Teletherapy	461	50.4 [45;50.4]	50.4 [45;50.4]	0.144
Brachytherapy	517	28 [21;28]	28 [21;28]	0.006
Total dose	518	73 [71.4;78.4]	73 [66;78.4]	0.125
Vaginal stenosis				
Teletherapy	459	50.4 [45;50.4]	50.4 [45;50.4]	0.746
Brachytherapy	515	28 [21;28]	28 [21;28]	0.088
Total dose	516	74 [71.4;78.4]	73 [66;78.4]	0.144

*p-Value: Mann-Whitney test.*

Table 3 presents a comparison between stenosis and the use of a vaginal dilator and the categorical variables studied. The comparison between the proportions of maintaining sexual intercourse and using a vaginal dilator was significant (p <0.001). This result shows that sexually active women use the dilator less, when compared to those who are not sexually active, contradicting professional recommendations for use for an indefinite period, regardless of whether or not sexual intercourse is maintained.

**Table 3:** Comparison between maintaining sexual intercourse, stenosis and the use of a vaginal dilator. Florianopolis (S.C.), Brazil, 2021

	Stenosis			Use of the vaginal dilator		
Maintenance of sexual intercourse	Yes (n=183) %(n)	No (n=333) %(n)	p-Value	Yes (n=255) %(n)	No (n=261) %(n)	p-Value
Yes No No information	28.80(64) 40.30(118) † 1	71.20(158) † 59.70(175) 0	0.009 †	39.80(88) 56.80(167) † 0	60.20(133) † 43.20(127) 1	<0.001

*p-Value: Chi -square test; t categories with positive local association are underlined.*

The description of proportions of the crossover between frequency of use of the vaginal dilator and the degree of stenosis was found in the records of 267 women (Table 4).

**Table 4:** Description of proportions of the crossover between frequency of use of the vaginal dilator and the degree of stenosis. Florianopolis-SC, Brazil, 2021

Weekly frequency of vaginal dilator use	Total (n=267) %(n)	Grade 0 (n=203) %(n)	Grade 1 (n=37) %(n)	Grade 2 (n=19) %(n)	Grade 3 (n=8) %(n)	p-Value
0	5.99 (16)	3.45 (7)	2.70 (1)	31.58 (6)	25.00(2)	0.001
1	9.74 (26)	9.36 (19)	8.11 (3)	15.79 (3)	12.50(1)	
2	35.21(94)	37.93 (2)	29.73 (11)	26.32(5)	12.50(1)	
3	49.06(131)	49.26 (100)	59.46 (22)	26.32(5)	50.00(4)	

*p-Value: Chi -square test*

Analyzing the associations between the frequencies of vaginal dilator use and the degree of vaginal stenosis, it was observed that most women did not present stenosis (Grade 0) and 49.10% used the dilator three times a week (131 women) (Table 4). Added to this finding is the percentage of use of the vaginal dilator and maintenance of sexual intercourse (Table 5). From the results, it was observed that 24.50% of women (127) do not maintain the use of vaginal dilation and sexual intercourse. However, dilator adhesion is close to 50%.

**Table 5:** Percentage of use of vaginal dilators and maintenance of sexual intercourse*. Florianopolis-SC, Brazil, 2021

Variable (n=519)	%(n)
Use of the vaginal dilator (three records without information) Maintenance of sexual intercourse (a medical record without information) Maintenance of sexual intercourse and associated use of the vaginal dilator Use of the vaginal dilator without maintaining sexual intercourse Maintaining sexual intercourse without using a dilator Non-maintenance of sexual intercourse and non-adherence to the use of the dilator	49.10(255) 42.80(222) 16.90(88) 32.10(167) 25.60(133) 24.50 (127)

Regarding the reasons for non-adherence to the use of the vaginal dilator, records were found in 152 medical records. Among these records, 35.53 of the women (54) justified their non-adherence by maintaining sexual intercourse after the end of brachytherapy; 17.11% (26) reported shame/ embarrassment or felt humiliated by the need to use the device; 7.89% (12) did not think it was necessary; 7.24% (11) justified the recent completion of treatment; 4.61% (7) due to lack of guidance on use, 4.61% (7) due to not having the device; 3.95% (6) due to fear, 1.97% (3) due to discouragement, 1.97% (3) due to children always being present; 1.32% (2) due to infection (urinary/candidiasis), 1.32% (2) due to forgetfulness; 0.66% (1) due to insecurity, 0.66% (1) due to disgust and 0.66% (1) due to bleeding.

Table 6 describes the changes in vaginal touch reported by women; 45.60% women (337) did not report complaints, 34.10% (117) had no records in the medical records, 9.40% (50) reported discomfort.

**Table 6:** Description of the variable changes in vaginal touch. Florianópolis (S.C.), Brazil, 2021

Changes in vaginal touch (n=519)	%(n)
No problem	45.60 (237)
Discomfort	9.40 (50)
Pain	4.20(22)
Burning	2.10 (11)
Bleeding	1.50 (8)
Pain + Bleeding	0.60 (3)
Pain + Discomfort	0.60 (3)
Discomfort + Bleeding	0.60 (3)
Discomfort + Burning	0.40 (2)
Pain + Burning	0.20 (1)
Pain + Bleeding + Burning	0.20 (1)
Pain + Discomfort + Bleeding	0.20 (1)
No information	34.10 (177)

## Discussion

The incident topographies found do not differ from international findings, pointing to cervical cancer as the most common (around 604 thousand estimated cases/year), followed by cancer of the uterine body, with emphasis on endometrial cancer (around 417 thousand estimated cases/year). However, in this study, the number of cases of vaginal cancer was higher than that of ovarian cancer, contradicting global epidemiology that points to the opposite order^(10)^.

Given these results, it is worth remembering that control of cervical cancer is a global public health priority and its control is more effective in developed countries. Incidence and mortality rates in low- and middle-income countries demonstrate ineffective control of a preventable disease. To control it, health resources and infrastructure are needed, coping with cultural barriers, use of technologies and the ability to adopt prevention and treatment strategies, articulating the three levels of care and prevention^11^.

Thus, the importance of all components of the health team and the role of the nurse associated with the applicability of guidelines for the control of cervical cancer stand out, considering the number of professionals existing in the health care network and proximity to women in the different services they seek for themselves and their families. Another role that is considered essential is that of health information and education, which must occur on an ongoing basis, in addition to formal education, as higher levels of education favor access to health.

The results obtained indicate an association between gynecological cancers and education, age and staging. More than half of the sample has been in school for a few years and is close to 50 years old. Adding these findings to those of another study^12^, which records the highest percentage of deaths in the 50-54 age group, it is clear that health actions must adopt specific measures for this age group.

Contradictorily, it was identified that almost 30% of women were under 40 years old. Another study also showed that the majority of women were up to the age of 39^13^. Therefore, it is highlighted that it is urgent to implement actions that change this reality. They are young women, who may have their lives ended early due to a diagnosis of an advanced disease or may live with a series of disabilities (infertility, sexual and psychological changes, urinary and fecal incontinence, lymphedema, among others), unknown to the majority of the population. and health professionals themselves, far from the oncology context. And for women of all ages, the staging found shows the risk of poor prognoses.

Study^14^ points out that the most important prognostic factor is the stage of the disease, with survival ranging from 80% to 95% for stage I disease, reducing to 16% for stage IVA disease. Another study^11^^)^ states that there is overwhelming evidence in developed countries that reductions in morbidity and mortality from cervical cancer can be achieved through the implementation of well-organized programs that aim to reduce the burden of disease and eliminate disparities social conditions, thus achieving shorter staging and less malignant morbidity.

Regarding vaginal cancer, the incidence found is highlighted, as well as the relationship between women's education and higher education and the predominance of women under 60 years of age. Given these results, ineffective health control can be deduced, but studies with greater territorial coverage would be necessary to better define this scientific evidence. These findings also differ from another study that points out that the disease is most commonly related to age over 60 and low levels of education^15^. In the national context (Brazil), women with vaginal cancer with primary education (40%) predominate^16^.

With regard to the total dose of radiation prescribed (67.9 to 74 Gy ), this result drew attention, as it may be contributing to gynecological toxicity, which in turn, influences the interruption of sexual activity and the degrees of stenosis found. Scientific evidence was not found discussing the relationship between the dose in Gy and the maintenance of vaginal stenosis, but they affirm genitourinary toxicity, and point out that most women reduce sexual activity or do not maintain sexual activity, because of sexual and psychological changes.

Other evidence^17^ states that severe genitourinary morbidity is the most frequently described complications by women undergoing pelvic radiotherapy. But although complete vaginal stenosis and mucosal ulceration are known to occur frequently after cervical cancer treatment, this morbidity is underreported. However, despite underreporting the study found a rate of vaginal stenosis in women with cervical cancer of 59% for Grade >1, 16% for Grade >2 and 1% for Grade >3. significant vaginal bleeding to Grade >2 post-treatment. More recent research^18^ reinforces the significant relationship between Grade >2 vaginal stenosis and the dose of ionizing radiation.

It is noteworthy that women with gynecological cancer are generally subjected to chemoradiation (teletherapy associated with chemotherapy, followed by brachytherapy), as experienced by the participants in this study, and the toxicities experienced are consequent to the sum of the effects of the therapies.

Analyzing the radiation dose received by the study sample, firstly, it is presented that the *American Brachytherapy Society* (ABS) recommends for the treatment of locally advanced cervical cancer a target dose (teletherapy followed by BATD) of 80-90 Gy and, 25-30 Gy in 4-5 separate fractions (5 fractions of 5.5 Gy each) with a maximum of two fractions per week and never on consecutive days^19^. In the sample studied, the total dose administered was equal to or lower than the ABS recommendation, what differed was the number and dose for each BATD session, as the scenario administers 3-4 sessions (7 Gy each) with the total dose varying 21 -28 Gy.

However, researchers^19^ who analyzed the dosimetric predictors of adverse events in radiotherapy in women with cervical cancer submitted to doses recommended by the ABS, found an overall rate of genitourinary adverse events (Grade 1) of 23.30% and serious adverse events (Grade 3) of 7.1%. In view ofthese results, they carried out a multicenter study that highlighted the need for more rigorous cumulative dosimetric targets than those indicated in the current ABS guidelines, being <80 Gy for the bladder, <65 Gy for the rectum, <65 Gy for the recto-vaginal point and < 70 Gy to sigmoid and intestine. From this perspective, analyzing the total dose in Gy received by the women who make up the sample of this study, it is suggested to analyze this evidence, relating it to clinical practice and women's needs for better control of toxicities.

Regarding the use of vaginal dilators, there are no defined standards regarding the time and duration of use^7^. The use of dilators is recommended to be started approximately 15 days after the end of pelvic radiotherapy, with the aim of preventing the formation of adhesions on the walls of the vaginal mucosa. The exercise of vaginal dilation, beyond one year after completion of treatment, appears to reduce the risk of developing vaginal stenosis^2^^, ^^20^^, ^^21^. These indications are followed in the study setting.

Adherence to the use of the vaginal dilator found in the study scenario represents moderate adherence (49.10%), with use three or twice a week. Considering the nature of the current investigation (cross sectional study), adherence to the use of the dilator over time was not evaluated, however, it is understood that health education by nurses during treatment and by physiotherapists during follow up has contributed to adherence already achieved. Maintaining sexual intercourse strongly influences women's non-adherence to the use of the dilator during follow-up and contributes to the prevention of vaginal stenosis in the short term. However, considering that vaginal stenosis is generally a late toxicity, sexual intercourse will not always guarantee effective dilation. In this sense, the dilator acts as a complementary alternative.

Another relevant finding of this investigation is related to the number of women who do not use a dilator and do not have sexual intercourse, which suggests a high risk of late vaginal stenosis. Thus, the importance of health education in the prevention of vaginal stenosis is highlighted and face-to-face and distance approaches and the use of continued informative materials are suggested.

Researchers observed that sexual activity decreased during radiotherapy and increased one year after its completion, when compared to pre-treatment frequency (p <0.001). Sexual pleasure continually decreased during and after completion of pelvic radiotherapy (p =0.013) and was negatively influenced by greater vaginal stenosis (p =0.01)^22^.

A study that combined the use of a vaginal dilator with exercises for pelvic floor muscles in vaginal stenosis, in women undergoing radiotherapy treatment for cervical cancer, identified that four months after radiotherapy, most women (90.9%) maintained/increased the size of the vaginal dilator and was sexually active (81.8%)^23^. Regarding the findings of changes in vaginal touch, study^24^ showed pain during sexual intercourse, changes in sexual function, bleeding and vaginal discharge. Therefore, considering the risks of vaginal stenosis and other changes involving the genital system, the relevance of gynecological examination and evaluation and classification of vaginal stenosis periodically after BATD is highlighted.

In this care scenario, the best health care requires the joint work of nurses, physiotherapists and doctors, covering prevention, health education, with continuous assessment and treatment of complications, as gynecological conditions are directly related to vaginal stenosis, which affects women's quality of life. As A limitation of the study is the evaluation of outcomes at a single moment, as well as the incompleteness of data records in the medical records of some women.

## Conclusion

There is evidence of an association between social, clinical and gynecological conditions in women after the end of BATD. Moderate adherence to the use of the dilator (verified in a single moment) and the maintenance of sexual intercourse negatively influenced adherence to the use of the vaginal dilator to prevent stenosis.

Health education and oncological care actions during and after the end of BATD contribute to the results evidenced on the use of vaginal dilators and prevention of vaginal stenosis, but need to be intensified, considering that a significant percentage of women do not adopt the use of the dilator, nor does it maintain sexual intercourse.
